# Exploring Health-Related Quality of Life in Patients with Anal Fistulas: A Comprehensive Study

**DOI:** 10.3390/life13102008

**Published:** 2023-10-03

**Authors:** Tudor Mateescu, Lazar Fulger, Durganjali Tummala, Aditya Nelluri, Manaswini Kakarla, Lavinia Stelea, Catalin Dumitru, George Noditi, Amadeus Dobrescu, Cristian Paleru, Ana-Olivia Toma

**Affiliations:** 1 Department of General Surgery, “Victor Babes” University of Medicine and Pharmacy, Eftimie Murgu Square 2, 300041 Timisoara, Romania; tudor.mateescu@umft.ro (T.M.); lazarfulger@yahoo.com (L.F.); george.noditi@gmail.com (G.N.); dobrescu.amadeus@umft.ro (A.D.); 2 Doctoral School, “Victor Babes” University of Medicine and Pharmacy, Eftimie Murgu Square 2, 300041 Timisoara, Romania; 3 Department of General Medicine, K.S. Hegde Medical Academy, Nityanandanagar, Deralakatte, Mangaluru 575018, India; anju9899@gmail.com; 4 School of General Medicine, Sri Siddhartha Medical College, Tumakuru 572107, India; 5 Kamineni Institute of Medical Sciences, School of Medicine, Hyderabad 500001, India; 6 Department of Obstetrics and Gynecology, “Victor Babes” University of Medicine and Pharmacy, Eftimie Murgu Square 2, 300041 Timisoara, Romania; dumitru.catalin@umft.ro; 7 Department of Thoracic Surgery, “Carol Davila” University of Medicine and Pharmacy, Bulevardul Eroii Sanitari 8, 050474 Bucuresti, Romania; cpaleru@gmail.com; 8 Department of Dermatology, “Victor Babes” University of Medicine and Pharmacy, Eftimie Murgu Square 2, 300041 Timisoara, Romania

**Keywords:** quality of life, anal fistula, inflammatory bowel disease, perianal disease

## Abstract

Anal fistulas often cause significant impairment to patients’ health-related quality of life (HRQOL). This cross-sectional study aimed to compare the HRQOL between patients with anal fistulas with inflammatory bowel disease (IBD) and those without, hypothesizing significant differences in HRQOL scores between these groups. The secondary objectives were to identify specific aspects of life quality most affected and explore potential variables influencing HRQOL. The study was conducted at the Clinical Emergency Hospital “Pius Brinzeu” in Timisoara, Romania, using a convenience sample of 175 adult patients diagnosed with anal fistulas, stratified into IBD and non-IBD groups. Quality of life was evaluated at initial hospital admission and three months post-treatment using four questionnaires: SF-36, GIQLI, HADS, and the WHOQOL-BREF. Initial SF-36 scores were marginally lower in the IBD group, with mean physical and mental scores of 52.0 and 54.5, respectively. Both groups showed an improvement after intervention, but the mean difference was higher in the IBD group, with an increase of 1.1 in physical score. Initial GIQLI scores were significantly lower in the IBD group (110) compared to the non-IBD group (116). Post-intervention, the mean scores increased to 116 and 121, respectively. HADS scores suggested higher anxiety levels in the non-IBD group (7.5 vs. 6.1), although depression scores were similar. Post-intervention, anxiety scores decreased more substantially in the non-IBD group (−0.9 vs. −0.3). The WHOQOL-BREF scores were lower across all domains for the IBD group at the initial test (physical health: 12.4, psychological health: 14.9, social relationships: 14.4, environment: 13.0). Post-intervention, scores increased marginally in the IBD group (physical health: 12.7, psychological health: 15.9, social relationships: 14.1, environment: 13.8) but varied in the non-IBD group. HRQOL, as measured by multiple questionnaires, is impacted differently in anal fistula patients with and without IBD. These findings highlight the importance of a tailored approach to managing this patient population to improve their quality of life post-treatment.

## 1. Introduction

Anal fistulas are a serious and often painful condition that can significantly impact an individual’s daily life [1]. This condition, characterized by an abnormal, inflammatory tract between the skin and the anal canal, is associated with a spectrum of diseases, including cryptoglandular infections, cancer, and inflammatory bowel disease (IBD) [2,3]. The subsequent physical discomfort, psychological distress, and disruption in routine activities can significantly affect the quality of life of the patient [4].

The concept of health-related quality of life (HRQOL) extends beyond mere physical parameters and encompasses mental, emotional, and social functioning [5]. HRQOL has been recognized as a significant outcome measure in a wide variety of chronic illnesses, providing a comprehensive evaluation of disease impact on patients’ lives [6,7,8]. Anal fistulas, although common, have not been extensively studied from all perspectives, rendering a gap in our understanding of how the condition interacts with patients’ wellness.

Inflammatory bowel disease (IBD), comprising conditions such as Crohn’s disease and ulcerative colitis, frequently presents with anal fistulas as a complication, such as perianal Crohn’s disease [9,10]. The co-occurrence of these conditions can compound the physical and psychological burden, potentially exacerbating the impairment of HRQOL. However, there is a lack of clarity on whether the presence of IBD in patients with anal fistulas translates to a differential impact on their HRQOL when compared to those without IBD.

While several studies have investigated the quality of life in patients with IBD [11,12], the focus on HRQOL specifically in patients with anal fistulas, with or without concomitant IBD, is limited [13,14]. Research has predominantly concentrated on clinical outcomes and management strategies, whereas the nuanced impacts on HRQOL, a patient-centric measure, are still a topic of interest [15,16]. Moreover, given the chronic nature of anal fistulas and their potential for recurrence, understanding the intricacies of HRQOL in this context becomes increasingly crucial. This comprehensive understanding is instrumental in improving patient-centered care and developing targeted interventions aimed at not only treating the condition but also enhancing the overall quality of life.

Therefore, the present study aims to examine the HRQOL in patients with anal fistulas, stratified based on the presence or absence of IBD. The study hypothesizes that there is a significant difference in the HRQOL between patients with anal fistulas with IBD and those without. Our primary objective was to compare and analyze these two patient groups’ quality of life scores, with secondary objectives including the identification of specific aspects of life quality most affected and the exploration of potential variables influencing HRQOL in these patients. 

## 2. Materials and Methods

### 2.1. Study Design

A cross-sectional study was designed at the Clinical Emergency Hospital “Pius Brinzeu” in Timisoara, Romania, following the guidelines of the Declaration of Helsinki. Background and medical data were obtained from the hospital database and the associated patients’ paper records, where all treatments, procedures, and demographics were registered.

The inclusion criteria required patients to be at least 18 years old with a confirmed diagnosis of anal fistula. These patients were divided into two groups. The first group comprised patients with anal fistulas related to IBD, while the second group consisted of patients with anal fistulas due to other causes. All patients with IBD were diagnosed after colonoscopy. Patients were excluded if they had incomplete medical records, a lack of consent identified from the personal paper records, a previous diagnosis of psychiatric disorders, or active colorectal cancer, which can also cause anal fistulas. Fistulotomy was performed for all included patients. 

A total of 200 patients were surveyed, of which 175 were included in the final analysis. Using a convenience sampling method, this sample size was determined. Considering the prevalence of anal fistula with an estimated margin of error of 5% and a confidence level of 95%, this sample size was deemed sufficient. The threshold for statistical significance was set at 0.05, and the statistical power (1-β) calculation was 80% for a type I error rate of 5%.

Two groups of patients were enrolled to investigate the impact of IBD on health-related quality of life in patients with anal fistulas. The first group consisted of patients with anal fistulas associated with IBD. The second group included patients with anal fistulas due to other causes, such as cryptoglandular infection, trauma, radiation, or obstetric injury, but not IBD. The anal fistulas were further classified according to the new classification (NC) as intersphincteric, transsphincteric, suprasphincteric, and extrasphincteric [17], and further into four grades (I, II, III, and IV). A simple fistula was considered for grade I + II, and a complex fistula was considered for grade III + IV + V. A low fistula involved less than one third of the external sphincter, while the higher fistula involved more than 1/3, according to the NC.

In the first group of 94 patients with IBD-related fistulas, the proportion of underlying diseases was as follows: 65% had Crohn’s disease, 30% had ulcerative colitis, and 5% had indeterminate colitis. In the second group of 81 patients with non-IBD fistulas, the causes were as follows: cryptoglandular infection (60%), trauma (15%), radiation (10%), and obstetric injury (15%). All patients were recruited from the same tertiary care hospital, and their diagnoses were confirmed by radiologic imaging, including magnetic resonance imaging (MRI), endoanal ultrasound, or computed tomography (CT) scan. The two groups of patients were matched by age and gender to avoid confounding factors that might alter the quality of life.

### 2.2. Study Instruments

This study undertook the task of evaluating the quality of life of the patients at two distinct points in time: during the initial hospital admission and three months post-treatment. The assessment of HRQOL and stress-related disorders in the patient cohort was performed using four distinct questionnaires: SF-36 [18], GIQLI [19], HADS [20], and the WHOQOL-BREF [21].

The Short Form-36 (SF-36) is a universally accepted questionnaire that appraises the health-related quality of life (HRQOL) and functional capabilities of an individual. With 36 items probing into eight different aspects of HRQOL, it provides an extensive overview of physical functioning, role limitations because of physical ailments, pain perception, general health perspectives, vitality, social functioning, role limitations due to emotional difficulties, and mental health. Respondents self-report their health status for the preceding month on a scale of 0 to 100, where a higher score corresponds to an improved health condition and quality of life. The summary scores can be categorized into the physical component summary (PCS) and the mental component summary (MCS) to convey that both the initial scores and the mean differences in scores were analyzed as part of our approach in scrutinizing the SF-36 data. We believe that this two-pronged analysis method adds depth to our study, enabling a more comprehensive understanding of the patients’ trajectories over the course of the study.

The gastrointestinal quality of life index (GIQLI) is an HRQOL questionnaire designed to gauge the influence of gastrointestinal diseases on an individual’s life-quality. The 36-item GIQLI encompasses five domains: gastrointestinal symptoms, physical function, emotional function, social function, and medical treatment, and it is scored on a 7-point Likert scale, with the total score ranging from 0 to 144. A higher score denotes a better health-related quality of life.

The hospital anxiety and depression scale (HADS) is a self-administered scale employed to identify anxiety and depression symptoms in individuals undergoing treatment in hospital or outpatient environments. With 14 items divided equally into assessing anxiety (HADS-A) and depression symptoms (HADS-D), it provides a comprehensive view of the patient’s mental state. The HADS purposely excludes questions on physical symptoms to avoid confusion between physical illness and psychological distress.

The WHOQOL-BREF is a well-known, abbreviated version of the World Health Organization’s quality of life assessment (WHOQOL-100). It was developed to provide a shorter but equally reliable and valid version of the original 100-item instrument. The WHOQOL-BREF contains 26 items that cover four domains: physical health, psychological health, social relationships, and environment. It also includes two items from the overall quality of life and general health facet of the original WHOQOL-100. Each item is rated on a scale of 1 to 5, with higher scores indicating better quality of life. This instrument is commonly used to assess the quality of life in various health conditions and different cultural settings. It was used to complement the data from the other questionnaires (SF-36, GIQLI, HADS) in the current study by providing a more comprehensive view of the patients’ overall perception of their quality of life, including aspects such as environment and social relationships that are not as thoroughly assessed in the other questionnaire. 

### 2.3. Statistical Analysis

All statistical analyses were performed using the SPSS v.26 for Windows (IBM Inc., Armonk, NY, USA). Normality of data was evaluated using the Kolmogorov–Smirnov test. For normally distributed data, central tendency and dispersion were represented by mean and standard deviation, respectively. The independent samples *t*-test was utilized to compare the means of the two groups under study. Non-normally distributed data, depicted in box plots, were characterized using median and interquartile range (IQR), and the Mann–Whitney U test was applied for comparisons between these variables. For categorical variables, frequencies and proportions were used. Comparisons between proportions were carried out using Fisher’s exact test as the assumptions for the chi-square test were not met. A *p*-value less than 0.05 was considered to indicate statistical significance in all analyses.

## 3. Results

### 3.1. Background Analysis

The background analysis incorporated 94 patients with inflammatory conditions and 81 with non-inflammatory conditions as shown in Table 1. The analysis examined various aspects such as age, gender, area of residence, smoking status, obesity, bowel habits, and Charlson comorbidity index (CCI) score. The inflammatory group had a mean age of 46.1 ± 11.3 years, whereas the non-inflammatory group had a slightly higher mean age of 48.3 ± 10.7 years. However, this difference was not statistically significant (*p* = 0.189). The age ranges for the inflammatory and non-inflammatory groups were 25–57 years and 31–64 years, respectively. In terms of gender, females constituted 46.8% and 48.1% of the inflammatory and non-inflammatory groups, respectively. The difference in gender proportion between these groups was not statistically significant (*p* = 0.859).

The area of residence was categorized as urban for 53.2% of the patients in the inflammatory group and 50.6% of those in the non-inflammatory group, with no significant difference between the two (*p* = 0.734). In the inflammatory group, 8.5% of the patients were smokers compared to 17.3% in the non-inflammatory group. This difference approached significance but did not meet the typical threshold for statistical significance (*p* = 0.080). Obesity was identified in 10.6% of the patients in the inflammatory group, significantly lower than the 22.2% observed in the non-inflammatory group (*p* = 0.037). Bowel habits differed significantly between the two groups as well, with 29.8% of patients in the inflammatory group experiencing frequent diarrhea compared to 16.0% in the non-inflammatory group (*p* = 0.032). Conversely, frequent constipation was more common in the non-inflammatory group (24.7%) than in the inflammatory group (7.4%) (*p* = 0.002). Lastly, a Charlson comorbidity index score greater than 3 was observed in 9.6% of the patients in the inflammatory group and in 18.5% of those in the non-inflammatory group. The difference approached significance but did not meet the typical threshold (*p* = 0.086).

### 3.2. Disease Characteristics

Table 2 delineates the characteristics of the disease among the two patient cohorts, specifically in terms of the position of the fistula, its complexity, and its location relative to the external sphincter. In terms of fistula position, 11.7% of the inflammatory group had intersphincteric fistulas, and 17.3% of the non-inflammatory group did, although this difference was not statistically significant (*p* = 0.292). Transsphincteric fistulas were found in 21.3% of the inflammatory group and 19.8% of the non-inflammatory group (*p* = 0.803), while suprasphincteric fistulas were identified in 30.9% and 24.7% of the respective groups (*p* = 0.365). The presence of extrasphincteric fistulas was similar in both groups: 36.2% in the inflammatory group and 38.3% in the non-inflammatory group (*p* = 0.774).

In terms of fistula complexity, a significant difference was observed between the two groups. The inflammatory group had a higher proportion of patients with complex fistulas (19.1%) compared to the non-inflammatory group (7.4%), and this difference was statistically significant (*p* = 0.024). Conversely, simple fistulas were more prevalent in the non-inflammatory group (92.6%) than in the inflammatory group (80.9%). Regarding the location of the fistula relative to the external sphincter, the non-inflammatory group had a higher proportion of patients with low fistulas (86.4%) compared to the inflammatory group (72.3%). This difference was statistically significant (*p* = 0.022). Meanwhile, high fistulas were more common in the inflammatory group (27.7%) than in the non-inflammatory group (13.6%).

### 3.3. Questionnaire Analysis

Table 3 depicts the analysis of the SF-36 questionnaire, which measures health status and quality of life, with higher scores indicating better outcomes. The table presents data from both the initial test and the post-intervention stage, in addition to the mean difference between these two stages. At the initial test, the mean physical score in the inflammatory group was 52.0 ± 7.1, which was slightly lower than that of the non-inflammatory group, with a mean score of 54.5 ± 7.7; this difference was statistically significant (*p* = 0.026). However, no significant differences were observed in the mental score (*p* = 0.624) or the total score (*p* = 0.209) between the two groups at the initial stage. Post-intervention, the physical, mental, and total scores all improved in both groups, with the mean physical score increasing to 53.1 ± 6.6 in the inflammatory group and 55.0 ± 6.8 in the non-inflammatory group (*p* = 0.062). Although the mean mental score and total score also increased in both groups, the differences were not statistically significant.

**Figure 1:**
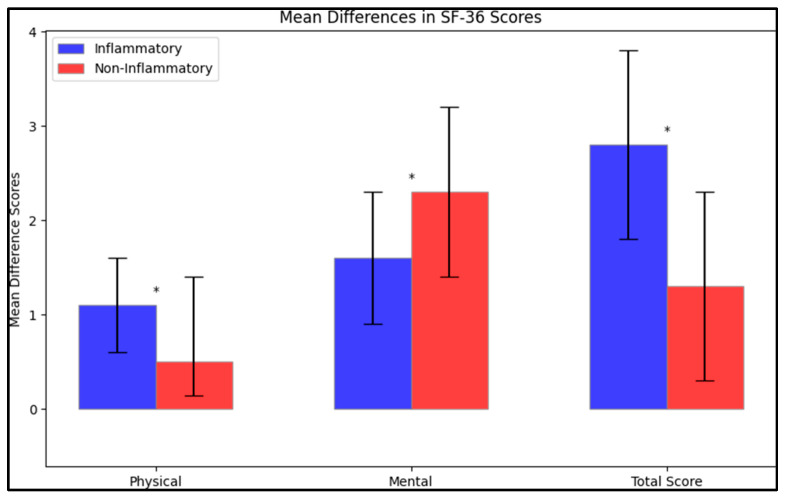
Mean difference on the SF-36 questionnaire after intervention; (*—Statistically significant differences at the 0.05 level of significance).

However, when looking at the mean difference in scores between the initial test and the post-intervention stage, the inflammatory group had a greater improvement in the physical score (mean difference of 1.1 ± 0.5) compared to the non-inflammatory group (mean difference of 0.5 ± 0.9) (Figure 1). This difference was statistically significant (*p* < 0.001). Similarly, the mean difference in the mental score was significantly higher in the inflammatory group (1.6 ± 0.7) than in the non-inflammatory group (2.3 ± 0.9), as was the mean difference in the total score (2.8 ± 1.0 in the inflammatory group vs. 1.3 ± 1.0 in the non-inflammatory group); both these differences were statistically significant (*p* < 0.001).

The Gastrointestinal Quality of Life Index (GIQLI) questionnaire results are presented in Table 4. Higher scores on the GIQLI indicate better health status and quality of life. Both the mean and median values were analyzed at two different times: at the initial test and post-intervention, with a comparison of the mean differences between these two stages. At the initial test, the mean GIQLI score of the inflammatory group was 110 ± 15.6, which was significantly lower than that of the non-inflammatory group, which had a mean score of 116 ± 13.2 (*p* < 0.001). The median GIQLI scores also showed a similar trend, with the inflammatory group having a median score of 112 (interquartile range, IQR: 99–125) and the non-inflammatory group having a median score of 114 (IQR: 104–124). The difference in the median scores was also statistically significant (*p* < 0.001).

Following the intervention, both the mean and median scores improved in both groups. The mean score in the inflammatory group increased to 116 ± 12.9, and in the non-inflammatory group, it increased to 121 ± 14.3. This difference remained statistically significant (*p* < 0.001). The median scores post-intervention were 118 (IQR: 103–133) and 120 (IQR: 109–131) for the inflammatory and non-inflammatory groups respectively, with the difference also being statistically significant (*p* < 0.001). The mean difference in scores between the initial test and post-intervention was calculated, revealing that the inflammatory group experienced a slightly larger mean increase (6 ± 2.7) compared to the non-inflammatory group (5 ± 1.1). This difference in mean change was also statistically significant (*p* < 0.001).

The results from the Hospital Anxiety and Depression Scale (HADS) questionnaire are detailed in Table 5. Higher scores on the HADS indicate greater levels of anxiety or depression. At the initial test, the inflammatory group had a mean anxiety score of 6.1 ± 4.3, which was significantly lower than the non-inflammatory group’s mean anxiety score of 7.5 ± 4.7 (*p* = 0.041). For the depression scores, the inflammatory group reported a mean score of 6.9 ± 3.8, which was higher than the non-inflammatory group’s mean score of 6.0 ± 3.5, but this difference was not statistically significant (*p* = 0.107). The total score, which combines anxiety and depression scores, was higher for the inflammatory group (12.4 ± 5.9) compared to the non-inflammatory group (11.3 ± 5.5), but this difference also lacked statistical significance (*p* = 0.206).

Following the intervention, both anxiety and depression scores decreased in both groups. The inflammatory group’s mean anxiety score went down to 5.8 ± 4.5, and the non-inflammatory group’s decreased to 6.6 ± 5.0, with this difference not being statistically significant (*p* = 0.267). The mean depression scores after the intervention were 6.7 ± 4.4 for the inflammatory group and 5.7 ± 3.6 for the non-inflammatory group, with the difference also not being statistically significant (*p* = 0.105). The total scores post-intervention were 11.4 ± 6.1 for the inflammatory group and 10.0 ± 5.2 for the non-inflammatory group. The difference in total scores post-intervention was also not statistically significant (*p* = 0.107).

**Figure 2:**
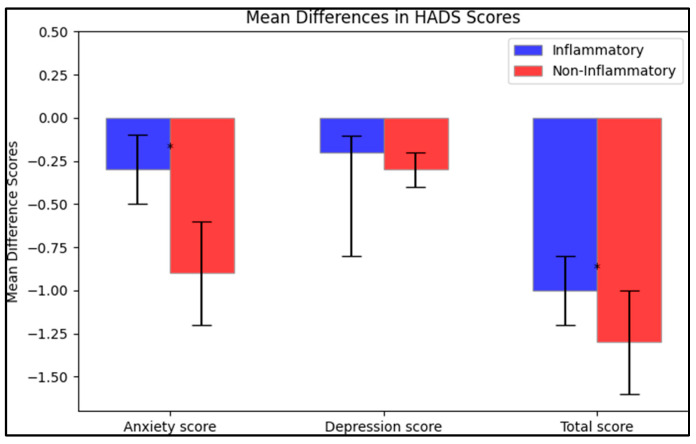
Mean difference on the HADS questionnaire after intervention; (*—Statistically significant differences at the 0.05 level of significance.

The mean differences between the initial test and post-intervention were calculated. In terms of anxiety scores, the inflammatory group saw a decrease of −0.3 ± 0.2, while the non-inflammatory group reported a more substantial reduction of −0.9 ± 0.3, with the difference between these two changes being statistically significant (*p* < 0.001). For the depression scores, both groups reported a slight decrease, with the inflammatory group having a mean change of −0.2 ± 0.6 and the non-inflammatory group a mean change of −0.3 ± 0.1; however, this difference was not statistically significant (*p* = 0.140). Finally, the total score mean change was −1.0 ± 0.2 for the inflammatory group and −1.3 ± 0.3 for the non-inflammatory group, and this difference was statistically significant (*p* < 0.001), as presented in Figure 2.

Table 6 presents the results from the World Health Organization quality of life (short version) or WHOQOL-BREF questionnaire. Higher scores in this questionnaire indicate better quality of life, with four domains explored: physical health, psychological health, social relationships, and environment. At the initial test, the inflammatory group reported lower mean scores across all domains when compared to the non-inflammatory group. The mean score for physical health was 12.4 ± 5.1 for the inflammatory group, which was significantly lower than the non-inflammatory group’s mean of 14.1 ± 5.8 (*p* = 0.040). Similarly, the inflammatory group’s psychological health mean score of 14.9 ± 5.6 was significantly lower than the non-inflammatory group’s mean of 17.3 ± 6.4 (*p* = 0.009). For the domains of social relationships and environment, the inflammatory group reported mean scores of 14.4 ± 4.2 and 13.0 ± 5.0 respectively, while the non-inflammatory group reported scores of 13.8 ± 5.0 and 13.9 ± 4.7. These differences, however, were not statistically significant (*p* = 0.389 and *p* = 0.223, respectively).

Following the intervention, the scores in the inflammatory group showed minimal improvements, while the scores in the non-inflammatory group decreased in the environment domain and increased slightly in the others. The post-intervention physical health score increased to 12.7 ± 3.8 for the inflammatory group, yet it was still significantly lower than the non-inflammatory group’s score of 14.9 ± 3.9 (*p* = 0.002). Similarly, the psychological health score for the inflammatory group increased to 15.9 ± 5.3, but remained lower than the non-inflammatory group’s score of 17.6 ± 5.1 (*p* = 0.032). The social relationships and environment domain scores for the inflammatory group post-intervention were 14.1 ± 4.9 and 13.8 ± 4.2 respectively, compared to 14.8 ± 6.0 and 12.2 ± 4.9 for the non-inflammatory group. These differences were not significant for social relationships (*p* = 0.369), but they were for the environment domain (*p* = 0.021).

**Figure 3:**
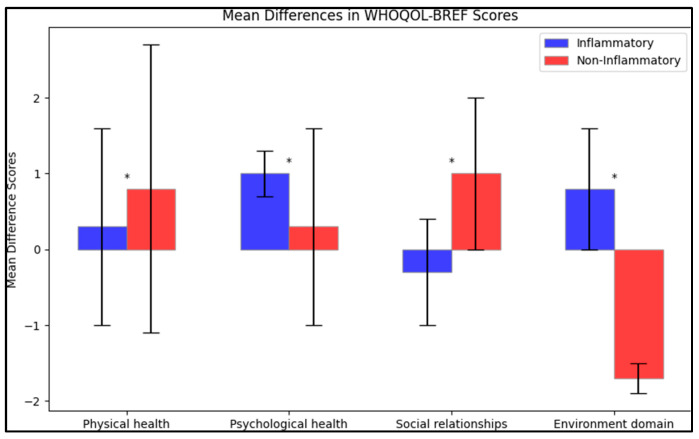
Mean difference on the WHOQOL-BREF questionnaire after intervention; (*—Statistically significant differences at the 0.05 level of significance.

In terms of mean difference between the initial test and post-intervention, there was an increase in the physical health score for both groups (0.3 ± 1.3 for the inflammatory group and 0.8 ± 1.9 for the non-inflammatory group), with the difference in change between the two groups being significant (*p* = 0.041). The psychological health score also increased for both groups, but the inflammatory group had a larger mean difference (1.0 ± 0.3 compared to 0.3 ± 1.3), with this difference being statistically significant (*p* = 0.001). For social relationships, the inflammatory group saw a slight decrease (−0.3 ± 0.7), while the non-inflammatory group saw an increase (1.0 ± 1.0), with the difference in changes being significant (*p* < 0.001). Finally, for the environment domain, the inflammatory group reported an increase (0.8 ± 0.8) while the non-inflammatory group reported a decrease (−1.7 ± 0.2), with the difference in these changes being statistically significant (*p* < 0.001), as presented in Figure 3.

## 4. Discussion

### 4.1. Literature Findings

Our study set out to examine the health-related quality of life (HRQOL) in patients suffering from anal fistulas, particularly focusing on the differences between those with inflammatory bowel disease (IBD) and those without. The data suggests that there is a significant variation in HRQOL between these two groups, confirming our hypothesis. Importantly, the quality-of-life scores were consistently lower for patients with IBD-related fistulas, as seen across multiple health and lifestyle domains in the SF-36, GIQLI, HADS, and WHOQOL-BREF questionnaires.

Our findings seem to agree with those of similar studies. For instance, a study by Casellas et al. [22] on the quality of life of patients with IBD found similar reductions in HRQOL across several domains, particularly in physical and mental health. Our results also echo the findings of a study by Kappelman et al. [23], which reported that patients with IBD have an impaired quality of life when compared to the general population, and that those with fistulas experience additional impairment. Additionally, our results indicate that the complexity of the fistula may also play a role in HRQOL. Patients with complex fistulas were more common in the inflammatory group, potentially contributing to their lower quality of life scores. This correlation was statistically significant (*p* = 0.024) and aligns with previous studies that suggest an increased complexity of disease is associated with reduced quality of life [24].

One of our secondary objectives was to identify specific aspects of life quality most affected in these patients. In our study, the physical and mental health domains in the SF-36 questionnaire were notably lower in the inflammatory group. Similarly, the WHOQOL-BREF questionnaire revealed that the physical and psychological health domains were significantly compromised in the inflammatory group, both before and after the intervention. These results provide valuable insight into the specific areas of HRQOL that are most affected, guiding targeted interventions to improve patients’ lives. In terms of changes after the intervention, we observed some notable differences. While scores improved across the board in the SF-36 and GIQLI questionnaires after intervention, the inflammatory group’s scores remained lower than those of the non-inflammatory group. This may suggest that interventions, while effective overall, may not fully address the unique challenges faced by patients with IBD-related fistulas.

Our study also showed that patients in the inflammatory group experienced significantly higher levels of anxiety, as shown in the HADS questionnaire. In regard to the apparent higher levels of anxiety in the non-IBD group, which might be seen as unexpected given the often-stressful nature of managing IBD, a potential explanation could be the other underlying causes of anal fistulas in the non-IBD group, such as trauma or obstetric injuries. These causes might be associated with higher initial anxiety levels due to the abruptness and acute nature of these conditions as opposed to the often-chronic course of IBD.

Further, the relatively higher depression score in the IBD group during the initial test might be reflective of the chronic and often debilitating nature of IBD, which might predispose these patients to higher levels of depression. This can be a focal point for further studies to delineate the psychological impacts of chronic vs. acute illnesses on patients’ mental health. The post-intervention results also reflected a decrease in both anxiety and depression scores across both groups, with the non-IBD group showing a more substantial decrease in anxiety levels compared to the IBD group. These dynamics can be a topic of further study, focusing on the differential impacts of interventions on mental health outcomes between these two distinct patient groups This finding is in line with previous research that indicates a higher prevalence of anxiety in patients with IBD [25]. It is important to highlight this as a significant consideration for treatment approaches and the overall management of IBD patients with fistulas. 

Moreover, our findings suggest that the HRQOL among patients with anal fistulas may be influenced by the fistula’s position relative to the external sphincter. The proportion of high fistulas was significantly higher in the inflammatory group (*p* = 0.022), potentially contributing to the lower quality of life in these patients. This observation is consistent with a study by Shirah [26] which indicated that fistula position can significantly impact HRQOL.

Global rates of incontinence following surgical treatment of anal fistula, wherein various medical materials are used for seton insertion, reportedly can reach up to 60% [27], while the recurrence rates can go up to 20% [28]. Comparatively, our study did not report the rates of incontinence among all participants, or the recurrence rate. A broad spectrum of incontinence rates has been reported in response to cutting seton treatment according to global literature, with Ritchie et al. [27] finding no correlation between incontinence, seton type, tightening frequency, or the fistula’s classification. Factors potentially influencing fistula recurrence could comprise the fistula’s complexity and height, presence or lack of a horseshoe extension, external opening’s laterality, the initial surgery’s technical failure in identifying the internal opening, and the surgeon’s overall experience in complex proctology [29].

Perianal fistula and its subsequent complications can considerably affect a patient’s quality of life, leading to various issues ranging from minor discomfort, poor hygiene, social embarrassment, to severe sepsis [30]. Our research participants from Saudi Arabia expressed significant satisfaction with their high healing rates and absence of incontinence following seton treatment. Notably, our study did not analyze the aforementioned potential factors contributing to fistula recurrence or the impact of successful treatment on religious practices. This may limit the generalizability of our findings and indicates a need for further research into these elements to better understand and manage anal fistula treatment outcomes.

Nevertheless, it should be noted that the differences between the IBD and non-IBD patients in terms of HRQOL can be attributed to the bowel habits, not only to the discomfort created by the anal fistulas. We observed in our study that patients with IBD had significantly more frequent diarrhea, thus this can influence the quality of life. In comparison, patients with anal fistulas and non-IBD had constipation problems significantly more often. This difference in bowel habits was previously described in other studies where it was observed that the major consequence of anal illness is a change in bowel habits, which has a substantial influence on the patient’s daily life and often results in severe physical and psychological problems [31,32].

Therefore, the current findings underscore the significant impact that anal fistulas, particularly those associated with IBD, have on a patient’s quality of life. Our study provides a comprehensive comparison of HRQOL between patients with anal fistulas with and without IBD, highlighting the physical and psychological challenges these patients face. These insights should guide the development of tailored treatment strategies to help improve quality of life in these patients.

### 4.2. Study Limitations

Our study has several limitations that should be noted. First, the study design was cross-sectional, which can establish correlation but not causality. Therefore, the findings should be interpreted with caution, particularly with regard to the associations between anal fistulas, IBD, and HRQOL. Second, all patients were recruited from a single tertiary care hospital, which could lead to selection bias and may limit the generalizability of our findings to other healthcare settings or geographical locations. The heterogeneity of care in different settings and regions might impact the HRQOL of patients with anal fistulas. This study was conducted in a region with a relatively homogeneous population, predominantly of white European ancestry. As such, the findings may not be directly translatable to populations with diverse racial backgrounds. Future multicentric studies involving a more racially diverse participant pool are necessary to explore potential racial variations in the presentation and severity of anal fistulas. This would enable a more comprehensive understanding and could pave the way for race-specific therapeutic approaches.

Third, our sample size was determined through convenience sampling, which, while sufficient for our study aims, might not fully represent the larger population of patients with anal fistulas, both with and without IBD. The non-random sampling method may also introduce bias into the study. Lastly, the study did not check for other potential confounding variables, such as lifestyle factors, socioeconomic status, and other co-existing medical conditions, which could have influenced HRQOL in our study population. Future studies should consider these factors when assessing HRQOL in patients with anal fistulas.

## 5. Conclusions

The current study serves as a preliminary exploration into the changes in health-related quality of life in patients with anal fistulas post-treatment, underscoring that patients with anal fistulas caused by IBD exhibit higher levels of stress and a lower quality of life than other patients with anal fistulas. Nevertheless, both the inflammatory and non-inflammatory groups saw improvements in health status and quality of life post-intervention, as indicated by various health questionnaires (SF-36, GIQLI, HADS, and WHOQOL-BREF). However, the inflammatory group demonstrated significantly greater gains in the physical, mental, and total scores on the SF-36, and a more substantial increase in the GIQLI scores compared to the non-inflammatory group. In contrast, while both groups recorded a reduction in anxiety and depression scores on the HADS post-intervention, the decrease was more pronounced in the non-inflammatory group. For the WHOQOL-BREF, the inflammatory group experienced minimal improvements in all domains except for the environment domain where an increase was noted, while the non-inflammatory group showed slight improvements in most domains but a decrease in the environment domain. The findings indicate marginal improvements in various domains within a three-month period. However, a longer follow-up is warranted to comprehensively understand the trajectory of quality-of-life improvements, especially in patients with IBD where achieving remission can be more prolonged.

## Figures and Tables

**Table 1:** Background analysis.

Variables	Inflammatory (*n* = 94)	Non-Inflammatory (*n* = 81)	*p*-Value
Age (mean ± SD)	46.1 ± 11.3	48.3 ± 10.7	0.189
Age range	25–57	31–64	–
Gender (female, %)	44 (46.8%)	39 (48.1%)	0.859
Area of residence (urban, %)	50 (53.2%)	41 (50.6%)	0.734
Smoking status (yes, %)	8 (8.5%)	14 (17.3%)	0.080
Obesity (yes, %)	10 (10.6%)	18 (22.2%)	0.037
Bowel habits (frequent diarrhea, %)	28 (29.8%)	13 (16.0%)	0.032
Bowel habits (frequent constipation, %)	7 (7.4%)	20 (24.7%)	0.002
CCI > 3 (*n*, %)	9 (9.6%)	15 (18.5%)	0.086

SD—standard deviation; CCI—Charlson comorbidity index.

**Table 2:** Characteristics of disease among the two patient cohorts.

NC	Inflammatory (*n* = 94)	Non-Inflammatory (*n* = 81)	*p*-Value
Position			
Intersphincteric	11 (11.7%)	14 (17.3%)	0.292
Transsphincteric	20 (21.3%)	16 (19.8%)	0.803
Suprasphincteric	29 (30.9%)	20 (24.7%)	0.365
Extrasphincteric	34 (36.2%)	26 (38.3%)	0.774
Complexity			0.024
Simple fistula	76 (80.9%)	75 (92.6%)	
Complex fistula	18 (19.1%)	6 (7.4%)	
Relative to external sphincter			0.022
Low fistula	68 (72.3%)	70 (86.4%)	
High fistula	26 (27.7%)	11 (13.6%)	

NC—new classification (of perianal fistula).

**Table 3:** Analysis of SF-36 questionnaire.

SF-36 (Mean ± SD)	Inflammatory (*n* = 94)	Non-Inflammatory (*n* = 81)	*p*-Value
Initial test			
Physical	52.0 ± 7.1	54.5 ± 7.7	0.026
Mental	52.5 ± 8.2	53.1 ± 7.9	0.624
Total score	52.9 ± 9.0	54.6 ± 8.8	0.209
Post-intervention			
Physical	53.1 ± 6.6	55.0 ± 6.8	0.062
Mental	54.1 ± 7.5	55.4 ± 7.0	0.240
Total score	55.7 ± 8.0	55.9 ± 7.8	0.867
Mean difference *			
Physical	1.1 ± 0.5	0.5 ± 0.9	<0.001
Mental	1.6 ± 0.7	2.3 ± 0.9	<0.001
Total score	2.8 ± 1.0	1.3 ± 1.0	<0.001

SF-36—36-Item Short Form Survey; higher scores indicate better health status and quality of life; SD—standard deviation; *—comparison between the mean difference between the initial test and after three months.

**Table 4:** Analysis of GIQLI questionnaire.

GIQLI (Mean ± SD)	Inflammatory (*n* = 94)	Non-Inflammatory (*n* = 81)	*p*-Value
Initial test			
Mean (±SD)	110 ± 15.6	116 ± 13.2	<0.001
Median (IQR)	112 (99–125)	114 (104–124)	<0.001
Post-intervention			
Mean (±SD)	116 ± 12.9	121 ± 14.3	<0.001
Median (IQR)	118 (103–133)	120 (109–131)	<0.001
Mean difference *			
Mean (±SD)	6 ± 2.7	5 ± 1.1	<0.001

GIQLI—The gastrointestinal quality of life index; higher scores indicate better health status and quality of life; SD—standard deviation; IQR—interquartile range; *—comparison between the mean difference between the initial test and after three months.

**Table 5:** Analysis of HADS questionnaire.

HADS (Mean ± SD)	Inflammatory (*n* = 94)	Non-Inflammatory (*n* = 81)	*p*-Value
Initial test			
Anxiety score	6.1 ± 4.3	7.5 ± 4.7	0.041
Depression score	6.9 ± 3.8	6.0 ± 3.5	0.107
Total score	12.4 ± 5.9	11.3 ± 5.5	0.206
Post-intervention			
Anxiety score	5.8 ± 4.5	6.6 ± 5.0	0.267
Depression score	6.7 ± 4.4	5.7 ± 3.6	0.105
Total score	11.4 ± 6.1	10.0 ± 5.2	0.107
Mean difference *			
Anxiety score	−0.3 ± 0.2	−0.9 ± 0.3	<0.001
Depression score	−0.2 ± 0.6	−0.3 ± 0.1	0.140
Total score	−1.0 ± 0.2	−1.3 ± 0.3	<0.001

HADS—hospital anxiety and depression scale; higher scores indicate greater levels of anxiety or depression; SD—standard deviation; *—comparison between the mean difference between the initial test and after three months.

**Table 6:** Analysis of the WHOQOL-BREF questionnaire.

WHOQOL-BREF (Mean ± SD)	Inflammatory (*n* = 94)	Non-Inflammatory (*n* = 81)	*p*-Value
Initial test			
Physical health	12.4 ± 5.1	14.1 ± 5.8	0.040
Psychological health	14.9 ± 5.6	17.3 ± 6.4	0.009
Social relationships	14.4 ± 4.2	13.8 ± 5.0	0.389
Environment domain	13.0 ± 5.0	13.9 ± 4.7	0.223
Post-intervention			
Physical health	12.7 ± 3.8	14.9 ± 3.9	0.002
Psychological health	15.9 ± 5.3	17.6 ± 5.1	0.032
Social relationships	14.1 ± 4.9	14.8 ± 6.0	0.369
Environment domain	13.8 ± 4.2	12.2 ± 4.9	0.021
Mean difference *			
Physical health	0.3 ± 1.3	0.8 ± 1.9	0.041
Psychological health	1.0 ± 0.3	0.3 ± 1.3	0.001
Social relationships	−0.3 ± 0.7	1.0 ± 1.0	<0.001
Environment domain	0.8 ± 0.8	−1.7 ± 0.2	<0.001

WHOQOL-BREF—World Health Organization quality of life (short version); SD—standard deviation; physical health, psychological health, social relationships, and environment; higher scores indicate better quality of life; *—comparison between the mean difference between the initial test and after three months.

## Data Availability

Data available on request.
